# Microstructure and Mechanical Properties of a New TWIP Steel under Different Heat Treatments

**DOI:** 10.3390/ma17092080

**Published:** 2024-04-28

**Authors:** Jiaruiming Zhang, Yu Bai, Wenxue Fan, Guanghe Zhang, Wenhui Zhang, Yang Yang, Hai Hao

**Affiliations:** 1 Key Laboratory of Solidification Control and Digital Preparation Technology (Liaoning Province), School of Materials Science and Engineering, Dalian University of Technology, Dalian 116024, China; zhangyuanxiang@mail.dlut.edu.cn; 2 Ningbo Research Institute of Dalian University of Technology, Ningbo 315016, China; fanwenxuedl@163.com; 3 Zhejiang Ruitai Suspension System Technology Co., Ltd., Ningbo 315500, China; hyl171129@163.com (G.Z.); yyc_30@163.com (W.Z.); 4 Ningbo Branch of China Academy of Ordnance Science, Ningbo 315103, China; yynbbky@126.com

**Keywords:** alloying, heat treatment, grain size, nano-precipitation, mechanical properties

## Abstract

The effects of solution treatment and annealing temperature on the microstructure and mechanical properties of a new TWIP steel that was alloyed from aluminum (Al), silicon (Si), vanadium (V), and molybdenum (Mo) elements were investigated by a variety of techniques such as microstructural characterization and room tensile testing. The austenite grain size grew slowly with the increase in annealing temperature. The relatively weak effect of the solution treatment and annealing temperature on the austenite grain size was attributed to the precipitation of MC and M_2_C, which hindered the growth of the austenite grain. The plasticity of the TWIP steel in cold rolling and annealing after solution treatment was obviously higher than that in cold rolling and annealing without solution treatment. This was because the large-size precipitates redissolved in the matrix after solution treatment, which were not retained in the subsequently annealed structure. Through cold rolling and annealing at 800 °C after solution treatment, the prepared steel exhibited excellent strength and plasticity simultaneously, with a yield strength of 877 MPa, a tensile strength of 1457 MPa, and an elongation of 46.1%. The strength improvement of the designed TWIP steel was mainly attributed to the grain refinement and precipitation strengthening.

## 1. Introduction

At present, lightweight, energy-saving, and emissions-reducing vehicles with high safety performance have become the inevitable trend in the development of new energy vehicles [1,2]. Since twinning-induced plasticity (TWIP) steel has both a high tensile strength and high plasticity, it exhibits significant application prospects in lightweight automotives [3,4]. The outstanding comprehensive mechanical properties result from several different deformation mechanisms, which are dislocation slip and deformation twinning. Deformation twinning is closely related to the low stacking fault energy values of TWIP steels. However, the characteristic austenitic microstructure of TWIP steels induces a lower yield strength in the range of 200–400 MPa, which is a technical bottleneck in the large-scale application of TWIP steels [5]. Therefore, it is an urgent problem to improve the yield strength of TWIP in order to ensure high plasticity. The widely recognized methods for improving the strength of TWIP steels include alloying and heat treatment [6,7]. Alloying is the addition of alloying elements to TWIP steels to improve the strength through solid solution strengthening and precipitation strengthening. Heat treatment is a way to improve the strength of TWIP steel by fine-grain strengthening via cold rolling and annealing [8,9,10,11,12].

Up to now, the researchers have developed several series of TWIP steels, which are classified into three generations [13]. The first generation of TWIP steels is Fe-Mn-Si-Al TWIP steel with a single austenite phase, which is attributed to the high Mn, Si, and Al contents. This type of high manganese steel exhibits higher plasticity, but the yield strength is relatively low, and its tensile strength is moderate. Because of the high content of Al and Si elements, the flow properties are poor, and there are certain problems in the casting and plating process. In order to solve the above problems, the researchers have developed second-generation TWIP steels with a composition of Fe-Mn-C. The strength is significantly improved due to its high content of C. However, there are also problems such as lower yield strength and hydrogen-induced delayed cracking. Therefore, the third-generation TWIP steel, which was alloyed with Al, Nb, V, Ti, Mo, and other elements on the basis of Fe-Mn-C is proposed. Some studies have shown that TWIP steel can achieve a good combination of strength and plasticity through the alloying of the above elements.

Lee et al. [14] added Si to Fe-18Mn-0.6C TWIP steel. When 1.5 wt.% Si was added, the yield strength was increased to higher than 500 MPa, and the tensile strength was increased to higher than 1200 MPa after annealing, but the plasticity was significantly reduced. By adding Al to Fe-Mn-C TWIP steel, it was found that the grain was refined, and the yield strength was increased, but the tensile strength was decreased [15]. When micro-alloying elements such as Nb, V, and Mo were added to TWIP steel, the micro-alloying elements were precipitated in the form of nanoscale carbide. The dispersed precipitates improved the strength of the material by hindering the movement of dislocation [16]. Moon et al. [17] added Mo to Fe-Mn-C-Al steel and found that the age-hardening behavior was changed due to the formation of κ-carbide precipitation, which increased the strength of steel but also reduced the plasticity. Razavi [18] and Nasajpour et al. [19] added about 1 wt.% Mo to TWIP steel to increase the yield strength from 200 MPa to higher than 400 MPa after cold rolling and annealing. V and Mo were simultaneously added to Fe-Mn-C-Al steel. Although the yield strength was increased to 785 MPa, the elongation was only about 20% [20]. Scott et al. [21] found that the strength of annealed and cold-rolled steel was not significantly improved (about 30 MPa) when 0.25 wt.% Nb was added to Fe-22Mn-0.6C. Compared with the addition of Nb, when adding 0.2 wt.% V to Fe-22Mn-0.6C, V carbides (VCs) mainly precipitated in the annealing process, and the size of the precipitates was smaller than Nb carbides. The yield strength and tensile strength were increased by 150 MPa and 100 MPa after annealing, respectively. The increase in strength was greater than that of Nb; meanwhile, the adverse effect on plasticity was less than that of Nb due to the small precipitate size. According to the above research, it can be seen that only adding one alloying element to TWIP steel makes it challenging to improve the strength and maintain high plasticity at the same time. Therefore, this paper intends to add multiple reinforcement elements such as Al, Si, V, and Mo to Fe-22Mn-0.6C TWIP.

In addition, heat treatment is an effective method for tailoring the balance of strength and elongation. Escobar et al. [22] studied the microstructure and mechanical properties of Fe-22Mn-0.45C TWIP steel at different annealing temperatures. It was found that the hardness of the TWIP steel increased with the rising cold deformation and decreasing annealing temperature. Therefore, the strength of TWIP steel can be improved by controlling annealing temperature and cold rolling deformation. In order to improve the plasticity of the TWIP steel, solid solution treatment was conducted [23]. When the solution temperature increased from 900 °C to 1150 °C, the yield strength of Fe-24Mn-0.5C-3.4Cr-0.3Mo-0.2Si after solid solution treatment decreased from 334 MPa to 317 MPa, but the elongation increased to 78%. When the temperature of the solution treatment is too high, the grain size becomes coarse. Although the plasticity is improved, the yield strength is reduced. The improvement of plasticity is not obvious when the solution temperature is too low. Therefore, the temperature of the solution treatment needs to be accurately controlled through a phase diagram. This paper attempted to improve the yield strength, tensile strength and elongation of Fe-Mn-C steel by alloying and adjusting the heat treatment process.

## 2. Materials and Methods

### 2.1. Experimental Materials

A typical high-strength TWIP steel Fe-22Mn-0.6C was selected as a basic material, and then JMatPro software (version 13.0) was utilized to optimize the chemical composition of TWIP steel. In the simulation, the general steel database and the quench properties tool in the phase temperature module were selected. The microstructure of the sample after quenching can be simulated by analyzing the phase diagram of adding different alloy components. Since the phase content in the microstructure is different with different added contents of various alloying elements, combined with the properties of each phase drawn from separate material databases, the mechanical properties of the materials can be predicted according to the phase composition. Therefore, it was used to calculate the influence of Al, Si, Mo, and V elements on the tensile strength, yield strength, and elongation of TWIP steel. The optimized composition of the new TWIP steel was obtained, and the specific alloy composition is shown in Table 1.

### 2.2. Experimental Procedures and Methods

**Figure 1:**
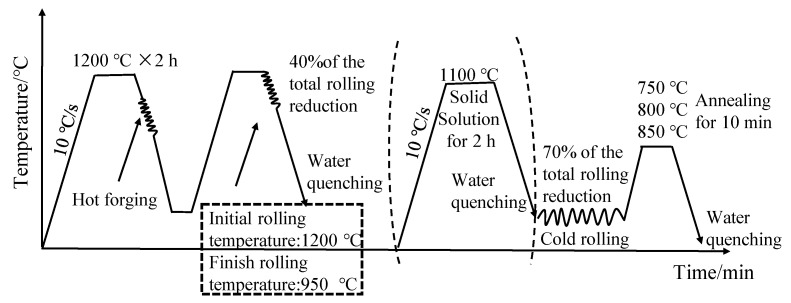
Flow chart of preparation experiment.

The designed alloy was melted by a ZG-50 melting furnace and casted into a round bar with a diameter of 80 mm and a length of 200 mm. The ingot was heated at 1200 °C and held for 2 h, then hot forging and hot rolling were carried out to obtain a plate with dimensions of 280 mm × 112 mm × 30 mm. The hot rolling process with the total reduction of 40% was conducted with an initial rolling temperature of 1200 °C and a final rolling temperature of 950 ℃. Then, different solution treatments were carried out: one was cold-rolled directly after hot rolling, and the other one was cold-rolled after holding at 1100 °C for 2 h followed by water cooling. The specimens are hereafter referred to as non-solid-solution-treated and solid-solution-treated, respectively. A ZK-NS9B rolling mill was used for multiple-pass cold rolling, with a rolling reduction of 70%. The cold-rolled samples were annealed at 750 °C, 800 °C, and 850 °C for 10 min, respectively, in a DZ47-60 heat treatment furnace, followed by water quenching. The experimental flow is shown in Figure 1.

The samples were electrolytically polished for 20 s in a mixed solution of 10% (volume fraction) HClO_4_ + 90% ethanol under an electrical voltage of 30 V. A SU5000 scanning electron microscope (SEM) was used to observe the microstructures. Two sizes of precipitates were observed in the microstructure. One is a large precipitate with a size of several hundred nanometers, which can be observed in SEM, and the precipitated phase is named as the large-size precipitate. The other is a precipitate of about ten nanometers observed in the transmission electron microscopy (TEM), which was named the small-size precipitate. The scale tool of the SEM software (version 13.0) was used to measure the large-size precipitates. A JEOL JEM-2100F (TEM) was used to analyze the small-size precipitates, which were extracted by the carbon extraction replication technique. For the crystallographic analysis, the microstructures were observed by IT800-SHL SEM equipped with electron back scatter diffraction (EBSD). The phases after annealing at different temperatures were analyzed using a D8 Advance Bruker X-ray diffractometer with a Cu target and a scanning range of 20–90°.

**Figure 2:**
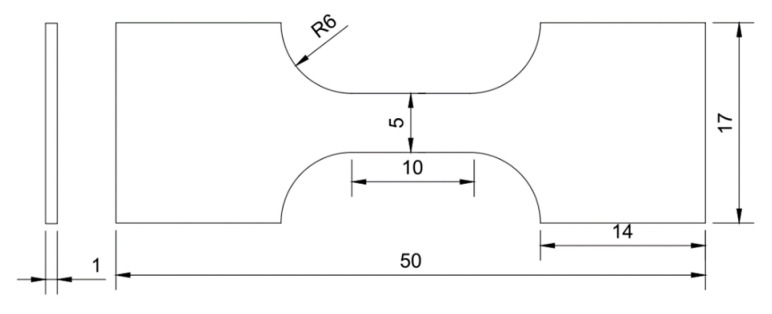
Illustration of tensile test specimen.

The tensile test samples were cut out from the cold-rolled plate along the rolling direction, and the size was referred to GB/T 228-2002 [24]. The tensile test specimen size is shown in Figure 2. After annealing at different temperatures, the tensile test samples were tested at room temperature by a CMT4304-DZ tensile testing machine with a cross-head speed of 0.5 mm/min.

## 3. Results and Discussion

### 3.1. Determination of Alloy Composition Bases in Prediction of Mechanical Properties

**Figure 3:**
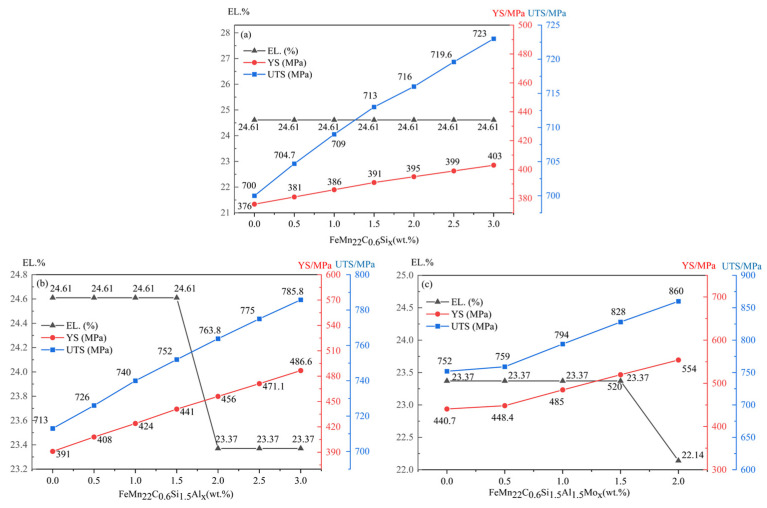
Variation in alloy strength and elongation with different element contents. (**a**) Fe-22Mn-0.6C-xSi; (**b**) Fe-22Mn-0.6C-1.5Si-xAl; (**c**) Fe-22Mn-0.6C-1.5Si-1.5Al-xMo.

Since the composition design of ultra-high-strength steel has a long research and development period, material design relying on calculation tools can reduce the number of tests and improve the efficiency of material research and development [25]. Therefore, in this paper, Java-based Materials Properties (JMatPro) software (version 13.0) was used to calculate the mechanical properties and phase diagram of the newly designed TWIP steels by adding Al, Si, Mo, and V elements. The brittle fracture occurs in TWIP steels with Si addition greater than 3 wt.%. It also affects the galvanizing quality of hot-rolled sheets [14,26]. Therefore, the addition amount of Si should usually be controlled within 3 wt.%. Figure 3a Fe-22Mn-0.6C-xSi shows the change in strength and plasticity with the increase in Si content. It can be observed that the increase in Si content in Fe-22Mn-0.6C TWIP steel improves the yield strength and tensile strength. The most significant increase in strength is observed when the Si addition is 1.5 wt.%. Meanwhile, the elongation is basically unchanged within 3 wt.% of Si content. Therefore, the addition of the Si element is determined as 1.5 wt.%.

Frommeyer et al. [27] found that when the content of Al in Fe-Mn-C steel exceeds 3 wt.%, it is easy to form an AlN inclusion and generate oxidation during casting [28]. Therefore, the amount of Al addition should be within 3 wt.%. On the other hand, it also prevents the delayed fracture and notch sensitivity of TWIP steel. The calculated results of the influence of Al content on the strength of the alloy are shown in Figure 3b. It can be seen that the yield strength and tensile strength are increased by about 30 MPa for every 1% content of Al. When the amount of Al is higher than 1.5 wt.%, the plasticity decreases significantly. Therefore, it is determined that the amount of aluminum is 1.5 wt.%, which can improve the strength and maintain the plasticity.

It has been reported that the addition of Mo with a mass fraction of 0.5% to 2% in high-manganese steel can effectively improve the strength of the casting [29]. The effect of Mo content on the strength and plasticity of the Fe-22Mn-0.6C steel is shown in Figure 3c. It can be seen that the strength of the alloy increases with the increase in Mo content. When the content of Mo is greater than 1.5 wt.%, the plasticity obviously decreases. Since the general amount of Mo added in the literature is about 1 wt.%, the added content of Mo is determined to be 1 wt.%. In the Fe-C phase diagram, when the V content exceeds 0.2%, the A_3_ point increases and austenitic stability decreases [30], so the added amount of V alloying is determined to be 0.2 wt.%.

According to the calculation results, the designed alloy composition Fe-22Mn-0.6C-1.5Si-1.5Al-1Mo-0.2V (wt.%) shows relatively good comprehensive mechanical properties. Hence, the newly studied TWIP steel with the above chemical composition is presented in this paper. The yield strength of the alloy steel is increased by 143 MPa, and the tensile strength is increased by 128 MPa compared with the Fe-22Mn-0.6C matrix.

### 3.2. Phase Diagram and Phase Content Calculation

**Figure 4:**
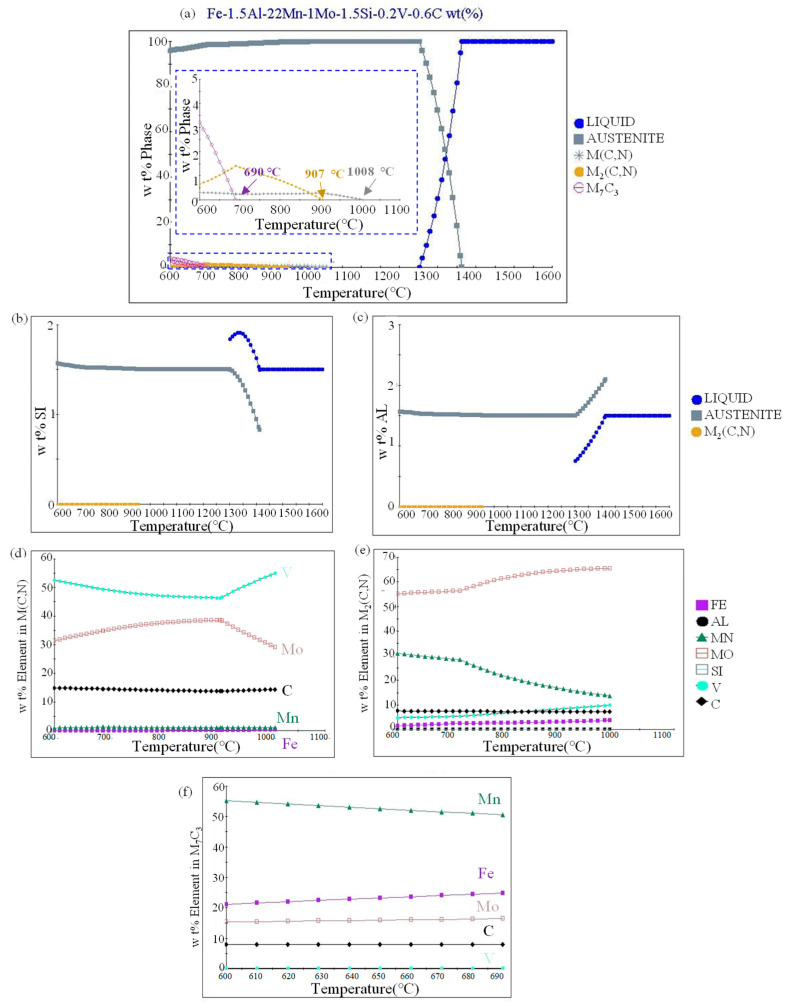
(**a**) Equilibrium phase diagram of Fe-22Mn-0.6C-1.5Si-1.5Al-1Mo-0.2V steel; (**b**,**c**) distribution of aluminum and silicon in different phases; (**d**–**f**) composition of three kinds of precipitated phases.

As shown in Figure 4a, the equilibrium phase diagram of the Fe-22Mn-0.6C-1.5Si-1.5Al-1Mo-0.2V steel was calculated by using JMatPro software (version 13.0). According to the phase diagram, the alloy begins to enter the solid phase zone at 1250 °C, and the matrix is all austenite at 1008–1250 °C. In addition, to ensure that carbides are stable at high temperatures and maintain a fine grain size, the temperature of the solid solution should not be too high. Therefore, 1100 °C was selected as the solution treatment temperature in this paper. In order to prevent excessive types and quantities of precipitated phases from reducing plasticity, 750 °C, 800 °C, and 850 °C were selected as the annealing temperatures in this paper, so that the microstructure could be a fine austenitic matrix with two kinds of precipitates. One precipitate is M(C,N) phase, which starts to precipitate at 1008 °C. The other precipitates is the M_2_(C,N) phase, which starts to precipitate at 907 °C. Figure 4b–d shows the distribution of elements in the phases M(C,N), M_2_(C,N), and M_7_C_3_, where M represents elements such as Fe, Mn, Mo, and V. M(C,N) represents carbides of V and Mo. M_2_(C,N) is a carbide of Mo, Mn, and V. M_7_C_3_ is a carbide of Mn, Fe, and Mo.

Figure 4e,f shows the distribution of Al and Si in different phases. It can be seen that 1.5 wt.% Al and 1.5 wt.% Si are almost solidly dissolved in the austenitic matrix. The Al and Si atoms enter the matrix crystal lattice, which distorts the crystal lattice and forms an elastic stress field. The stress field has a strong effect on the stress field around the dislocation, which causes the strengthening and enhancement of the yield strength of the material, as predicted in Figure 3.

### 3.3. Microstructure Evolution

**Figure 5:**
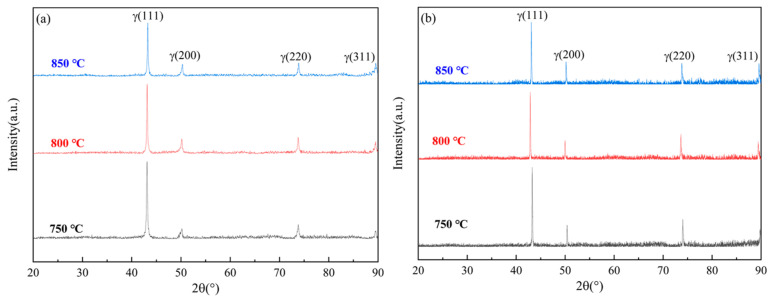
X-ray diffraction pattern of the new TWIP steel after cold rolling and annealing. (**a**) non-solid solution; (**b**) with solid solution treatment.

In order to explore the effect of solid solution treatment and different annealing temperatures on the microstructure of the new TWIP steel after cold rolling and annealing, XRD phase analysis was conducted. Figure 5 shows the X-ray diffraction pattern of the new TWIP steel after cold rolling and annealing under the conditions of non-solid solution and solution treatment. It can be observed that only one austenite phase (γ) peak is found in both samples, whether it is solution-treated or not. The absence of martensitic peaks in the XRD patterns indicates that a martensitic phase transformation has not occurred. Presumably due to the small content of the precipitated phase, a corresponding obvious diffraction peak was not found in the X-ray diffraction profiles. Therefore, the identification of the precipitated phase also required further characterization by SEM and TEM.

**Figure 6:**
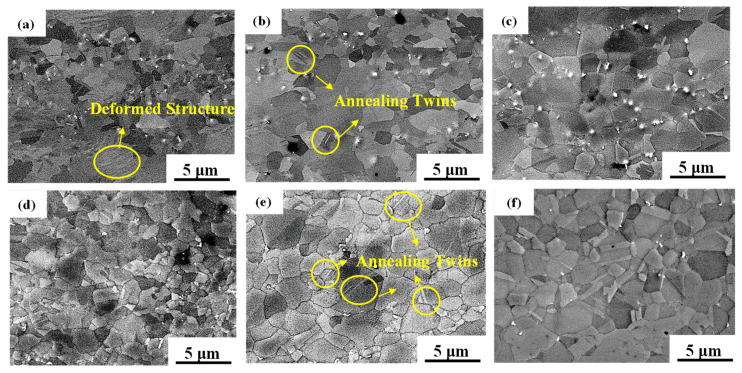
SEM images of the samples with different heat treatments. (**a**–**c**) Cold-rolled and annealed at 750 °C, 800 °C, and 850 °C for 10 min with non-solid solution treatment; (**d**–**f**) cold-rolled and annealed at 750 °C, 800 °C, and 850 °C for 10 min after solid solution treatment at 1100 °C for 2 h.

**Figure 7:**
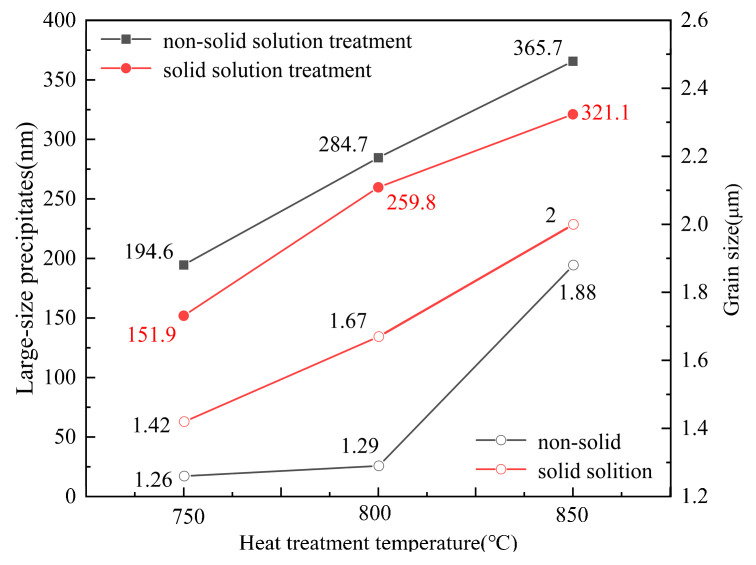
Size changes under different heat treatment processes.

Figure 6a–c shows the microstructures of samples annealed at 750 °C, 800 °C, and 850 °C for 10 min, respectively, without solid solution treatment before the cold rolling process. It can be seen that there are also a small number of deformed structures after annealing at 750 °C. This indicates that the sample was not fully recrystallized at 750 °C. Grains were completely recrystallized at 800 °C and 850 °C. At the 800 °C annealing temperature, there were a large number of annealing twins and some elliptical bright precipitate phases were distributed inside the grains and at the grain boundaries. In addition, it can be observed that the precipitates reflected a growing tendency when the annealing temperature increased to 850 °C; the growth of the grain size and precipitates was not obvious. The austenite grain size and precipitated phase size of the experimental steels were measured by the linear intercept method (and by counting the grain and twin boundaries), and the result is shown in Figure 7. The grain size rose from 1.26 μm to 1.88 μm and the large size of the precipitates grew by 90.1 nm when the annealing temperature increased from 750 to 850 °C. The large-size precipitates grew significantly, but the grain size obviously did not grow with the increase in annealing temperature.

Figure 6d–f shows the microstructures of the cold-rolled and annealed samples after 2 h of solid solution treatment at 1100 °C. It can be observed that the grain size tended to grow with the increase in annealing temperature, and the grain size increased from 1.42 μm and 1.67 μm to 2 μm. The size of the precipitates after solution treatment was obviously smaller than that in the non-solid solution (as can be seen in Figure 6). With an increase in annealing temperature from 750 °C to 850 °C, the increase in grain size between adjacent temperatures was small, approximately ~0.3 μm on average (see Figure 7), and the increase in the precipitated phase size between adjacent temperatures was approximately ~90 nm.

In general, the austenite grain size of Fe-Mn-C steels obviously increased with an increase in annealing temperature [31,32,33]. However, the grain growth of austenite was so large that even the annealing temperature interval was 50 °C. This should be attributed to the presence of nano-sized precipitates. In the annealing temperature range of 750–850 °C, there were nano-sized particles inside the grains as well as on the grain boundaries (Figure 6). These particles can effectively hinder grain boundary migration and reduce the grain growth rate during the annealing process, resulting in a smaller grain size and coarsening with the elevated temperature. Therefore, the grain size growth was not obvious with the increase in annealing temperature. After solid solution treatment at 1100 °C, the size of the precipitates was smaller than that in the non-solid-solution-treated sample (see Figure 7). The decrease in the size of the precipitates after solid solution treatment is due to the large-size precipitate phase being dissolved into the matrix during solid solution treatment, and then precipitates with smaller sizes forming again during annealing at lower temperatures and short annealing times.

As shown in Figure 6, small-size nanoscale precipitates can be faintly seen in the grain. The characterization of small-size precipitates under SEM is not clear, so TEM is used to observe the distribution of the small-size precipitate phase, morphology, and size.

**Figure 8:**
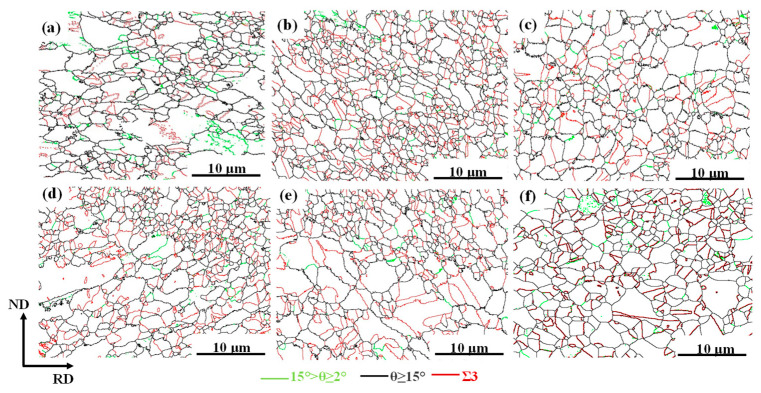
EBSD diagrams of grain boundary with different heat treatment processes. (**a**–**c**) Annealing at 750 °C, 800 °C, and 850 °C without solution treatment; (**d**–**f**) annealing at 750 °C, 800 °C, and 850 °C, respectively, under solution treatment at 1100 °C.

Figure 8 shows the EBSD grain boundary maps of the TWIP steel under different heat treatment conditions, where small-angle grain boundaries, large-angle grain boundaries, and twin boundaries are represented by green, black, and red, respectively. The twin boundary density data obtained by SEM showed that the fraction of annealing the twin boundary increased from 14% to 29% when the annealing temperature increased from 750 °C to 800 °C and decreased from 29% to 26% when the annealing temperature increased to 850 °C with solution treatment. In the condition of non-solid solution treatment, the twin density increased from 15% to 21% when the annealing temperature increased from 750 °C to 800 °C. When the annealing temperature increased to 850 °C, the twin boundary density decreased from 21% to 20%. The twin boundary density increased first and then decreased with the increase in annealing temperature. It reached the highest at 800 °C, regardless of the solution type. In addition, the density of the twin boundary after solution treatment was higher than that in the non-solid solution. A higher density of the annealing twin means a higher strength can be obtained.

**Figure 9:**
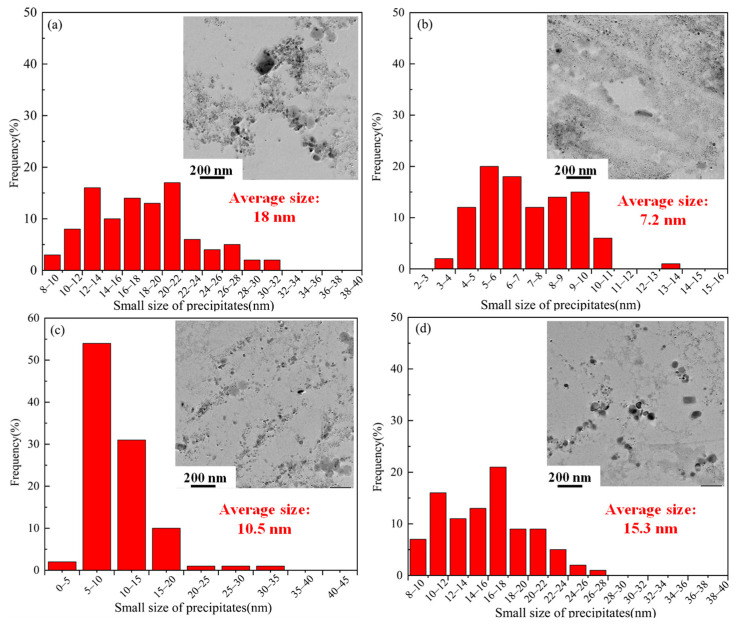
TEM images of the precipitated phase. (**a**) Non-solid solution and annealing at 800 °C; (**b**) solid solution and annealing at 800 °C; (**c**) solid solution and annealing at 750 °C; (**d**) solid solution and annealing at 850 °C.

In order to further study the small-size precipitates, the microstructures of the TWIP steel were observed by TEM through extraction phase analysis. Figure 9 shows the morphology, size, and distribution of the small-size precipitates in the matrix. It can be observed that the precipitates mainly existed in circle and oval shapes. As shown in Figure 9a, the precipitates were concentrated and agglomerated together in the specimen annealed at 800 °C without the solid solution. The average grain size of the precipitated phase is 18 nm in Figure 9a. Compared with the specimen without solid solution treatment, the size of the precipitated phase is obviously reduced and the distribution in the matrix is more uniform in the 800 °C annealed specimen with solid solution treatment. The average grain size of the precipitated phase is 7.2 nm in Figure 9b. Figure 9b–d shows the relationship between the size and distribution of the small-size precipitates as the annealing temperature increases. Compared with the concentrated distribution without the solid solution, the precipitated phase was distributed in a band under the conditions of the solid solution. Meanwhile, the size of the small-size precipitates decreased first and then increased as the annealing temperature increased. The size of the small-size precipitates was the smallest at 800 °C with the solid solution.

**Figure 10:**
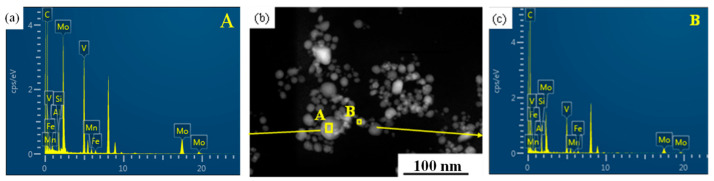
TEM images of morphology and energy spectrum. (**a**,**c**) energy spectrum of precipitates of point A and B, respectively, in (**b**); (**b**) TEM morphology of precipitates cold-rolled and annealed at 850 °C after solid solution treatment at 1100 °C.

Figure 10 shows the morphology and energy spectrum of the precipitates under TEM observation. It can be seen that the diffraction peak profiles of point A (the precipitated phase with a larger size) and point B (the precipitated phase with a smaller size) are different. The specific composition is shown in Table 2. Based on the elemental species of the precipitated phase calculated by the JMatPro software (version 13.0), it was determined that point A is the M_2_(C,N) phase, in which M mainly represents V, Mo and Mn elements. Point B is the M(C,N) phase, in which M mainly represents V and Mo elements. Since no nitrogen element was found in the scanning region, A is the M_2_C phase and point B is the MC phase.

As shown in Figure 6 and Figure 10, the precipitation phase in the cold-rolled and annealed new TWIP steel can be roughly divided into two categories: the first category is the large particles with sizes larger than 100 nm under the scanning electron microscope observation; the second category is the other composite precipitation of MC and M_2_C.

### 3.4. Effect of Solid Solution Treatment and Annealing Temperature on Mechanical Properties

**Figure 11:**
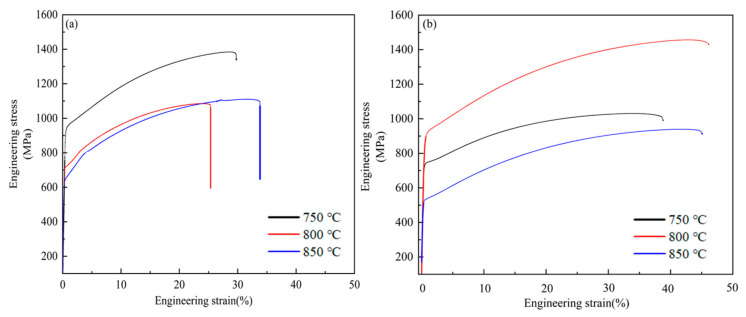
Engineering stress–strain curves of test steel under different heat treatment processes. (**a**) Engineering stress–strain curves of annealing at 750 °C, 800 °C and 850 °C for 10 min under non-solid solution condition; (**b**) Stress–strain curve of cold rolling and annealing with solid solution.

Figure 11 shows the engineering stress–strain curves of the new TWIP steel after cold rolling and annealing. The new TWIP steel shows a continuous yield without an obvious yield plateau during the tensile process. In Figure 11a, in the non-solid-solution-treated specimens, the yield strength decreases, and the tensile strength decreases first and then increases slightly with the increase in annealing temperature, but the tensile strength does not change significantly. The reason why the strength does not change significantly is that the grain size does not increase significantly with the increase in annealing temperature. In Figure 11b, in the specimens with solid solution treatment, the yield strength and tensile strength increases first and then decreases as the annealing temperature rises. The elongation of the specimens with solid solution treatment is significantly higher than that without solution treatment. The specific mechanical properties are shown in Table 3. A good balance between high strength and high plasticity is achieved when cold rolling and annealing at 800 °C for 10 min after solid solution treatment. The yield strength is higher than that of Fe-22Mn-0.6C TWIP, whose grain size is about 1 µm in the literature. Furthermore, its plasticity is not greatly affected [34]. This indicates that the modification of the chemical composition and preparation process enhanced the mechanical properties.

Generally, yield strength and tensile strength decrease monotonically, and elongation increases monotonically with increasing annealing temperature [35]. The fluctuation in the mechanical properties of the new TWIP steel in this experiment does not conform to the conventional law. It is known that the increase in flow stress of metallic materials is related to the following aspects: solid solution strengthening, dislocation strengthening, precipitation strengthening, and grain refinement. It is generally known that the increase in flow stress is the sum of the above strengthening mechanisms. In the present study, the chemical compositions were the same for all specimens, and the microstructures of the cold-rolled and annealed specimens were fully recrystallized so that the initial dislocation density was supposed to be the same. Thus, the solid solution strengthening and dislocation strengthening effect on yield strength enhancement did not make a difference for the specimens, whether solution-treated or not. The grain size and the amount of precipitated phase were changed by changing the annealing temperature and solid solution conditions. Therefore, the contribution of fine grain strengthening and precipitation strengthening to the flow strength was changed in the different specimens. Due to the grain size being refined to about 1.5 μm on average after cold rolling and annealing, the strength was greatly improved. However, the change in grain size was not obvious, whether the specimens were solid-solution-treated or not, and was followed by the same annealing condition. Therefore, the strength enhancement of the solid-solution-treated specimens was not related to the grain size, but probably related to the nanoscale precipitates.

According to the inference of Gladma [36], the increase in strength generated by the second phase particle is shown in Equation (1):

(1) $$∆YS_{p}=0.538Gb\left(\frac{{f-}^{\frac{1}{2}}}{X}\right)\ln\left(\frac{X}{2b}\right)$$

where G is the shear modulus in MPa, f is the volume fraction of the MC and M_2_C precipitates, and X is the mean particle diameter expressed in μm. From Formula (1), it can be found that increasing the volume fraction of precipitates and decreasing the average diameter of precipitates were conducive to increasing the contribution of precipitation strengthening in steel. In Figure 6, the size of the precipitates observed under SEM is larger than 100 nm. It not only can not improve the strength, but also cause a reduction in plasticity. The plasticity also decreases due to the increase in the number of large-size precipitates [37]. The size of the large-size precipitates under the condition of non-solid solution treatment was larger than that in the solid solution treatment; therefore, the plasticity with solid solution treatment was greater than that without solid solution treatment. The precipitates below 10 nm that were observed under TEM were fully involved in precipitation strengthening and ensured the strength and plasticity of the material, according to the Formula (1). In Figure 9b, when the annealing temperature is 800 °C, the size of precipitated phase is the smallest and the content of precipitates is the highest. The precipitates can effectively hinder the dislocation movement and strengthening of the matrix. They can also hinder grain boundary movement and increase the strength [38]. Furthermore, the higher annealing twin boundary density in the specimen annealed at 800 °C reduced the dislocation-free path as well as prevented dislocation movement, which also contributed to the enhancement of strengthening. Therefore, the strength was highest when annealing at 800 °C after solid solution treatment. The increase in the content of the M_2_C and MC phases was the main reason for the increase in the flow stress.

## 4. Conclusions

The effects of annealing temperature and solution treatment on the microstructure and mechanical properties of a new TWIP steel were studied. A heat treatment method was explored to improve the strength and ductility. After solid solution treatment at 1100 °C for 2 h, cold rolling, and annealing at 800 °C for 10 min, the new TWIP steel (Fe-22Mn-0.6C-1.5Si-1.5Al-1Mo-0.2V) showed the best comprehensive mechanical properties; the yield strength increased to 877 MPa. At the same time, the tensile strength increased to 1457 MPa, and the elongation reached 46.1%. Compared with Fe-22Mn-0.6C TWIP steel having a grain size of 2um, the yield strength of the TWIP steel after alloying and heat treatment in this paper increased by 447 MPa, and the tensile strength increased by 321 MPa, although the elongation somehow decreased [39]. The improvement of strength was mainly due to fine grain strengthening and precipitation strengthening. The grain size after annealing at 800 °C reached 1.67 μm; meanwhile, a large number of dispersed MC and M_2_C phases with sizes of about 10 nm that precipitated from the matrix hindered the dislocation movement and improved the strength.

## Figures and Tables

**Table 1:** Chemical composition of the newly designed TWIP steel. (wt.%).

Fe	Mn	Al	Si	Mo	C	V	S	P
Bal.	22	1.48	1.52	1	0.62	0.2	0.0161	0.0079

**Table 2:** TEM-EDS results (at.%) for the different phases in point A and B in Figure 10.

	C	V	Mo	Si	Mn	Fe	Al
A	75.3	12.4	8.5	2.8	0.6	0.4	0.1
B	89.5	4.7	3.0	2.5	0.1	0.2	-

**Table 3:** Mechanical properties data under different heat treatment conditions.

	Annealing Conditions	Yield Strength (MPa)	Tensile Strength (MPa)	Total Elongation (%)	Grain Size (μm)
Non-solid solution	750 °C 10 min	781	1154	29	1.26
	800 °C 10 min	739	1086	25.3	1.29
	850 °C 10 min	636	1110	33.8	1.88
Solid solution at 1100 °C	750 °C 10 min	729	1030	38.8	1.42
	800 °C 10 min	877	1457	46.1	1.67
	850 °C 10 min	523	939	45.1	2

## Data Availability

Data are contained within the article.
